# Phytomicrobiome communications: Novel implications for stress resistance in plants

**DOI:** 10.3389/fmicb.2022.912701

**Published:** 2022-10-06

**Authors:** Kanika Khanna, Sukhmeen Kaur Kohli, Nandni Sharma, Jaspreet Kour, Kamini Devi, Tamanna Bhardwaj, Shalini Dhiman, Arun Dev Singh, Neerja Sharma, Anket Sharma, Puja Ohri, Renu Bhardwaj, Parvaiz Ahmad, Pravej Alam, Thamer H. Albalawi

**Affiliations:** ^1^Department of Botanical and Environmental Sciences, Guru Nanak Dev University, Amritsar, India; ^2^Department of Microbiology, DAV University, Jalandhar, India; ^3^Department of Zoology, Guru Nanak Dev University, Amritsar, India; ^4^State Key Laboratory of Subtropical Silviculture, Zhejiang A&F University, Hangzhou, China; ^5^Department of Botany and Microbiology, College of Science, King Saud University, Riyadh, Saudi Arabia; ^6^Department of Botany, S.P. College Srinagar, Jammu and Kashmir, India; ^7^Department of Biology, College of Science and Humanities, Prince Sattam Bin Abdulaziz University, Al-Kharj, Saudi Arabia

**Keywords:** phytomicrobiome, rhizosphere, stresses, metabolomics, metagenomics, defense mechanisms, plant probiotics

## Abstract

The agricultural sector is a foremost contributing factor in supplying food at the global scale. There are plethora of biotic as well as abiotic stressors that act as major constraints for the agricultural sector in terms of global food demand, quality, and security. Stresses affect rhizosphere and their communities, root growth, plant health, and productivity. They also alter numerous plant physiological and metabolic processes. Moreover, they impact transcriptomic and metabolomic changes, causing alteration in root exudates and affecting microbial communities. Since the evolution of hazardous pesticides and fertilizers, productivity has experienced elevation but at the cost of impeding soil fertility thereby causing environmental pollution. Therefore, it is crucial to develop sustainable and safe means for crop production. The emergence of various pieces of evidence depicting the alterations and abundance of microbes under stressed conditions proved to be beneficial and outstanding for maintaining plant legacy and stimulating their survival. Beneficial microbes offer a great potential for plant growth during stresses in an economical manner. Moreover, they promote plant growth with regulating phytohormones, nutrient acquisition, siderophore synthesis, and induce antioxidant system. Besides, acquired or induced systemic resistance also counteracts biotic stresses. The phytomicrobiome exploration is crucial to determine the growth-promoting traits, colonization, and protection of plants from adversities caused by stresses. Further, the intercommunications among rhizosphere through a direct/indirect manner facilitate growth and form complex network. The phytomicrobiome communications are essential for promoting sustainable agriculture where microbes act as ecological engineers for environment. In this review, we have reviewed our building knowledge about the role of microbes in plant defense and stress-mediated alterations within the phytomicrobiomes. We have depicted the defense biome concept that infers the design of phytomicrobiome communities and their fundamental knowledge about plant-microbe interactions for developing plant probiotics.

## Introduction

A glaring overhaul has been observed in the 21st century in regard to climate change where environmental stresses have caused global threat toward food safety and security. The world population has been projected to be 9 billion (approx.) by 2050, and environmental disturbances cause reduced crop productivities and soil fertility thereby impeding agricultural sustainability (Muller et al., 2017; Chouhan et al., 2021). Consequently, it will likely enhance more than 60% of the food demand, specifically for the cereal crops (wheat, maize, and rice), leguminous crops, pulses (peas, lentils, and soybeans), etc., to feed the massive population (Majeed et al., 2017). The yields of these crops are often challenged with the poor soil and biotic and abiotic stresses (Suzuki et al., 2014; Kumar et al., 2021). Stresses cause a plethora of adverse effects onto plants in terms of morphology; biochemistry; and metabolic, physiological, and molecular reactions. To meet the ongoing demands of food, traditional agricultural practices are exclusively used in which the use of pesticides and fertilizers is most common, but they lead to polluted and contaminated ecosystem. Henceforth, in order to safeguard the agricultural sustainability and boost the crop yields for posterity, we need to adopt novel and environmental friendly methods (Majeed et al., 2017; Chouhan et al., 2021).

Since decades, enormous studies have depicted the sky-scarping diversity of microbiome co-linked to agricultural plants (Lemanceau et al., 2017). Microbiome holobiont refers to huge microflora associated with plants, and plant microbiome involves the microbial genomes in regard to host plants (Brader et al., 2017; Bhatt et al., 2020). Phytomicrobiome is found in various niches in the form of phyllosphere, endosphere, and rhizosphere in order to promote plant growth along with soil fertility (Vorholt, 2012; Etesami and Adl, 2020). Plant microbiome has opened the avenues for the usage of microbes as biofertilizers and biopesticides, and public interest has been considerably enhanced to use this biological resource as alternatives for external inputs toward agriculture. For, this phytomicrobiome interaction enables us to effectively understand the beneficial aspects of microbiome partners to be used as ecological engineers (Glick, 2012). Soil microbiologists have been trying to utilize microbes also called as inoculants for stimulating soil fertility and nutrition. For successful utilization, various trials are first done for regular practice. Phytomicrobiome contains numerous microbes, such as eubacteria, bacteria, fungi, archea, protozoa, virus, etc., that play an imperative role in soil-plant system (Theis et al., 2016).

Phytomicrobiome is the most sustainable and efficient approach that is effective both in terms of productivity and quality (Yadav et al., 2020). Harnessing the biological resources in agriculture improves both productivity as well as social outcomes in a constructive manner. This microbiome technology aids the beneficial plant-linked microbiomes toward enhancing quality and quantity of agriculture with minimal usage of resources along with plummeting environmental stresses (Kour et al., 2020). Microbiomes are basically host-linked microflora that inhabit different tissues onto host surfaces along with inter/intra colonization of host cells. As huge diversity of microbes is linked with plant roots, therefore, they can be used to alter the host metabolism, physiology, and defense systems. This, in turn, substantially induces the growth, yield, vigor, and efficiency of the plants for nutrient acquisition and stress tolerance (Kour et al., 2020). The mutualistic associations among plants and microbes benefit both the partners and microbiomes that help in shaping the host phenotype and act as a shield among host genotypes and environment (Rana et al., 2020). There are abundant of facts that support the plant microbiome and further revolutionize the agriculture sector. To elucidate, the integration of crops with effective management practices for enhancing productivities and the power of eradicating different pests and pathogens through microbes serve to be one of the positive aspect. Most importantly, this minimizes the chemical fertilizer usage and improves crop fitness with minimal interference of chemical stimulants (Kumar et al., 2019).

Rhizosphere, a niche of microbes surrounding plant roots stimulates a plethora of signaling processes of plants along with the exchange of different materials across the plant-microbial interface (Qiao et al., 2017). Among this zone, plant growth-promoting microbes hold the crucial rank in natural ecosystem and in the agriculture sector as it enables to boost the plant growth *via* nitrogen fixation, mineral acquisition, inhibiting pathogenic organisms and modulating plant defense responses toward stresses (Shameer and Prasad, 2018). There are a plethora of reports that have outlined the direct and indirect mechanisms of microbes in promoting crop yields with enhancing soil nutrition, nutrient uptake, and enhancing overall activities of plants (Qiao et al., 2017; Kumar et al., 2019). Different microflora have been found to improve soil quality, remediate heavy metal-contaminated soil, suppress pathogenic organisms through secreting antagonistic metabolites with enhanced immunity toward pathogens (Berendsen et al., 2018; Shameer and Prasad, 2018). More to the discussion, these beneficial microbes have attained a great reputation in managing various abiotic and biotic stresses. Henceforth, considering their overall beneficial aspects, they have been deployed as alternatives for yield enhancement and used as biofertilizers and inoculants in the fields for elevating the soil fertility and crop production. Keeping in view the prevailing situations, this review has been designed to discuss all the aspects in regard to phytomicrobiome. We have aimed toward exploring each and every facet of microbiomes and its predominant role against stresses. It is quite evident that the agricultural sector is facing the serious challenge in terms of yields and productivities, and, for maintaining its quality and quantity, this has been found to be the most economical and eco-friendly approach. Thus, global urge for a sustainable approach to meet the food security worldwide has led to develop amended and novel sustainable agricultural patterns.

## Phytomicrobiome: An interface between soil and plants

Under natural environmental conditions, plants are closely associated with a plethora of well- orchestrated and complex microbial community in the phytomicrobiome or the plant microbiome. These intimate associations generally involve the rhizosphere region, endosphere region, pollens, and nectar and leaf surfaces of plants, and are collectively termed as phytomicrobiomes. Under altering environments, these associations do not remain static in the phytomicrobiome, however, undergoes abrupt changes with respect to its structural composition as well as the functional activities of its host partners. (Meena et al., 2017; Li et al., 2018). Recent literature has inferred that these continuous oscillations in the microbiomes are not passively controlled by the host plants only, but it is an outcome of millions of years of co-evolution between the microbes and the host partners. This co-evolution likely triggers the plants to initiate active cooperation with these microbes under stressful environmental conditions (Barnawal et al., 2017; Xu et al., 2019; Morcillo and Manzanera, 2021). Plants, in particular, undergo an unusual phenomenon “cry for help” strategy, generally after experiencing a kind of abiotic or biotic stress. Furthermore, they are deployed by a plethora of microbial species through specific signaling molecules to amplify their potential to counter these stresses (Bhat et al., 2020). In general, plant and microbes interactions are critically important for the plant growth, nutrient acquisition, and shielding of multiple stressors (Kosová et al., 2018).

The plant-microbe interface generally includes the key hot spots for the plant microbe interactions, likely to be in the root region and rhizosphere. Plants release specific root exudates to attract a diverse group of microbes under stress conditions, and these exudates account for the 11 to 40% of the photosynthetically prepared organic carbon (Pandey et al., 2017; Ismail et al., 2021). They found that sugar beet roots under pathogen ‘*Rhizoctonia saloni’* exposure attract *Flavobacterium* and *Chitinophage* inside the endosperm to counteract these fungal pathogens. On the other hand, Ismail et al. (2021) found that leguminous plants intensify flavonoids secretions under nitrogen-limiting conditions to attract diverse groups of nitrogen-fixing microbes. While, the maize crops secrete certain phenolic compounds like benzoxazinoides to counteract the stresses, as a defense mechanism (Singhal et al., 2016; Wang et al., 2016). Biotic and abiotic stressors exhibit extreme losses in growth and productivity of food crops and thereby causing constraints toward global food security. Sivasakthi et al. (2014) reported that there is annual estimated loss in the yield of food crops of approximately USD$220 billion. However, it has also been reported that chemical control methods are not found to be environmentally friendly and economic; thus, biological tools have been recommended over the chemical control agents (Farooq et al., 2009). To achieve this, microbiome engineering is considered to be a sustainable and effective approach to minimize the yield losses and to improve overall productivity. Pandey et al. (2017) found that plant stress-related amino acids 1-aminocyclopropane-1-carboxylate (ACC) is capable of reshaping the soil microbiome composition under salinity stress and triggers the stress responses. Likewise, rhizosphere exhibits a plethora of signaling molecules contributed by plants and microbial partners that possess potential to counteract multiple stresses, therefore needed to be explored.

### Rhizosphere and microbiome alliance

#### Rhizospheric interactions

Rhizosphere is among the primary sites where microbial communities interact with their plant partners. Both the pathogenic and beneficial microbial communities reside in the rhizosphere region. Also their composition varies in response to the variation in the soil composition and its chemical properties (Pérez-de-Luque et al., 2017). Rhizobacterial partners in the rhizosphere trigger multiple responses, i.e., improve the soil nutrients, stabilize soil health, root growth, and further promote the remediation responses (Badri et al., 2009). This alliance and cooperation among the rhizobacteria and roots within the rhizosphere promotes the growth and proliferation of plant root system and improves the nutrient uptake toward the shoots (Traxler and Kolter, 2015; Zhang et al., 2017). However, microbial populations are found to enhance the crop yields and are used as alternatives to the chemical fertilizers in the field.

#### Root exudation

The rhizobacterial-rhizosphere interactions maintain the levels of root exudates secretions, which are required to attract the microbial populations (el Zahar Haichar et al., 2014; Semchenko et al., 2014). Root exudates act as mediators between the plant-microbe interaction and further impact roots colonization and root growth. These root exudates contain a wide range of organic compounds, i.e., peptides, sugars, vitamins, amino acids, enzymes, and a series of primary and secondary metabolites (Basiliko et al., 2012; Korenblum et al., 2020). Thus, the microbial populations correspond to a variety of root exudates constituents in the rhizosphere region, thus support the growth of beneficial microbial populations and improve the yield and biomass of different crop plants. Whereas, some root exudates inhibit the growth of harmful microbial flora and provide defensive action against them (Berlanas et al., 2019; Raklami et al., 2019). Baysal et al. (2013) assessed through proteomic studies that elimination of soil-borne pathogens occurred by the introduction of Bacillus species in the rhizosphere region. Bona et al. (2019) also carried out his studies to confirm the activities of different microbes in the rhizosphere region by applying the metaproteome approaches. In this approach, they conducted proteomic studies of the rhizosphere of *Vitis vinifera* and observed that the bacterial species associated with different groups like *Pseudomonas*, *Streptomyces*, *Bradyrhizobium*, *Bacillus*, and *Bulkhorderia* genus are found to have high-protein expressions and thus induce multiple regulatory processes in the rhizosphere region.

**FIGURE 1:**
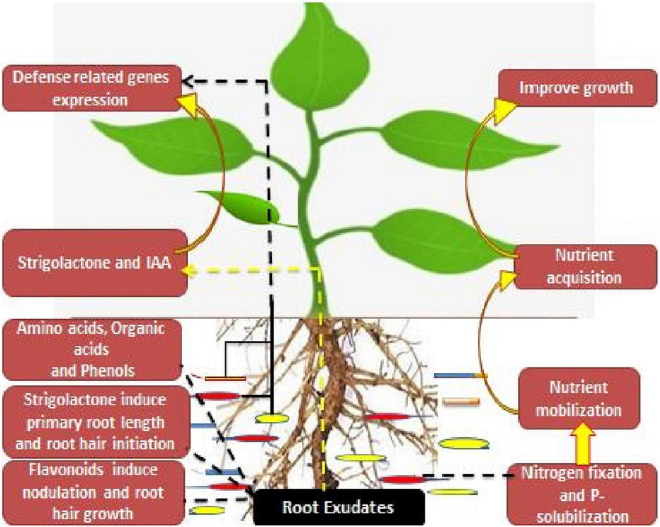
Depicting root/rhizosphere and microbiome alliance.

Strigolactone, a root exudate as well as a novel phytohormone, is found to induce primary root length as well as the root hair elongation in the rhizosphere region. They are released as root exudates in the rhizosphere *via* a plethora of dicot and monocot plants, where they mediate mutualistic associations between the root system and the arbuscular mycorrhizal fungi (De Cuyper et al., 2015). Also they participate in the symbiosis between the rhizobia and the leguminous crops (Yang et al., 2019). Among these root secretions, organic acids are found to have high-energy compounds for the microbial partners as well as mediate the regulation of different bio-geochemical cyclic secretions in the rhizosphere region (Wu et al., 2013). However, the low carbon compounds in the root exudates intricate the biosynthesis of rhizobacterial-associated phytohormones, whereas the tryptophan exudates act as a precursor for indole-3-acetic acid (IAA) biosynthesis (Li et al., 2018). Also, ACC, an ethylene precursor, is used as carbon and nitrogen sources for the microbial partners (Xiang et al., 2019). On the other hand, leguminous plants provide flavonoids as root exudates, which further mediate the transcriptional regulation of rhizobia Nod factors (NFs). Furthermore, these Nod factors participate in the nodulation initiation as well as the root hair initiation process (Figure 1).

These root-rhizobacterial associations in the rhizosphere region influence the defense-related mechanisms in plants under different environmental stresses. Rhizobacterial partners influence the regulation of stress-responsive genes, i.e., heat shock proteins, ascorbate peroxidase, and S-adenosyl-methionine synthetase (Gontia-Mishra et al., 2016). They also provide resistance against root herbivores and other diseases in the rhizosphere region (Maheshwari et al., 2015). These associations or alliance between the plants and the microbial partners assist the hosts in nutrient acquisition, growth promotion, and scaling up the yields (Rahimi et al., 2020). These intricate associations are of much importance for both plants and soil health (Wang et al., 2018; Anbi et al., 2020; Etesami and Adl, 2020).

#### Microbiome and root architecture

Microbes have the potential to effectively undergo colonization of the roots in order to stimulate their growth through direct and indirect methods. The modulation in the root architecture *via* root-kinked microbes involves the phytohormonal assembly, such as auxins, cytokinins, ethylene, and other signaling molecules that cause lateral root branching and root hair formation. After the microbial proximity toward plant roots, they tend to convert the exudates into plant hormones (Arora et al., 2020; Khan et al., 2020). There occurs the modulation in the root exudation process with the different developmental stages of the plants; thereby, rhizo-microbiome alignment varies accordingly (ALKahtani et al., 2020). They also possess antagonistic properties toward pathogenic organisms through releasing certain enzymes, antibiotics, siderophores, and crucial nutrients for improving the root architecture (Hartman and Tringe, 2019).

#### Secondary metabolites-mediated interactions

Plants face several stresses throughout their life time that hamper their physiological and metabolic activities, therefore need to be managed for their growth and survival. These stresses are directly related to microbes that offer them to understand and alter certain mechanisms to counteract such adversities. Many stress resistance mechanisms are related to microbes that aid modulation in the metabolic network of plants and help in the activation of stress-related metabolites and genes, respectively (de la Fuente Cantó et al., 2020). There is huge popularity of microbes synthesizing stress-related metabolites in current times. In addition, they also alter transcriptional machinery for stress resistance in plants. The upregulation of the ABA-signaling cascade also modulates the expression of *WRKY* and *MYB* during stresses (Gamez et al., 2019). A wide range of genetic alterations has been observed in plants in response to microbe-mediated alterations of their metabolic pools (Kousar et al., 2020). A myriad of secondary metabolites, namely, compatible solutes and volatile compounds have been found to be released within the rhizosphere that forms the basis for the microbial-mediated interactions. Different microbes possess traits for stress tolerance, osmoregulation at cellular levels, ionic homeostasis, reducing toxic ions (Kumar and Verma, 2018). Alongside, they also release various volatile compounds or anti-pathogenic metabolites, such as amino acids, sugars, polyols, betaines, etc., in order to tolerate harsh conditions (Kumar and Verma, 2018).

#### Plant-microbe interactions

Plant growth is considerably stimulated through the application of plant growth-promoting microbes as well as mycorrhizal fungi. There are different mechanisms (direct or indirect) that enhance the beneficial traits in plants during stresses. Various biochemical and molecular pathways are adopted by microbes for maintaining growth through hormonal regulation and nutrient acquisition, thereby enhancing the plant growth regulators for resistance against adverse conditions (Spence and Bais, 2015). As depicted earlier, microbes release an array of metabolites to reduce the incidence of pathogen attack and also facilitate certain metabolic processes, such as phosphate solubilization, nitrogen fixation, hormonal signaling, exopolysaccharide production, etc., to avoid the undesirable events (Złoch et al., 2016). Along with this, they possess the ability to synthesize crucial enzymes, namely, glucanases, ACC-deaminases, lytic enzymes, and chitinases, to counteract negative situations. Due to these factors, they may act as potent fertilizers as well as pesticides in agricultural practices. Followed by this, this may also improve the overall nutritional quality as well as yield, thereby prove to be a boon to farmers in terms of increasing the financial income and promoting organic and sustainable agriculture.

## Phytomicrobiome-mediated soil fertility, plant health, and growth

**FIGURE 2:**
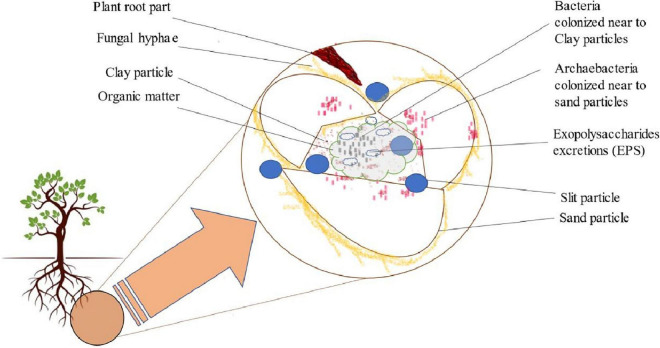
Diagrammatic presentation of phytomicrobiomes in soil aggregates.

Soil is a complex microbial biome whose community structure, composition, and diversity are mainly influenced by a wild range of physical, chemical, and biological variables of soil. Each gram of soil consisted of around 10^9^–10^10^ microbial cells with an estimated 10^4^–10^6^ species (Vieira and Nahas, 2005; Roesch et al., 2008). Soil structure formation depends upon the shape and composition of a slit, sand, and clay particle aggregates that further form macro/micropores, giving rise to soil ecosystem (Figure 2; Vadakattu and Germida, 2014). Thus, these mineral particles aggregates can form different microhabitats through migration of smaller bacteria inside them, whereas fungal hyphae migrate within macropores (Kim and Crowley, 2013). Additionally, a molecular investigation on soil clay particles aggregates by Kim and Crowley (2013) was done through the 16S ribosomal gene. They found that the area with more clay particles retains more moisture and possesses high bacterial population. However, more archae bacteria are drawn toward sand particles, which have a lower water retention capability (Figure 2). Between these soil particles, microorganisms form a cluster to secrete polysaccharides responsible for fixing colonies and generating a cementing material between them. They also contribute to building up organic matter in these areas (Pereira et al., 2021). Moreover, the fungal hyphae operate mechanically to connect the soil particles. Apart from the plant–microbe interactions within the rhizospheric zone, different microorganisms interact with one another in these soil aggregates, allowing the evolution of microbial population in this biosphere (Bhat et al., 2020).

Most of the nutrients present inside the soil are not accessible to plants. However, phytomicrobiomes presence inside the soil enhanced the accessibility of these nutrients to plants (Table 1). Many bacterial species like *Bacillus, Pseudomonas*, etc., and fungal species like *Aspergillus* and *Penicillium* lead to the production of organic acids and enzymes that solubilize phosphate, thus making it available to the plants (Sańchez et al., 2017). Additionally, these phytomicrobiomes immobilize phosphates inside the organic matter and serve as nutrient storage with quick release in the dissolved form for the plant (Braos, 2015). Some bacterial genera like *Azospirillum*, *Rhizobium*, as well as a few cyanobacteria, fix atmospheric nitrogen and convert this element into an accessible form for plants (Shridhar, 2012). These microorganisms, especially *Rhizobium*, are used as a bio-fertilizer and have been popular in soybean production, providing a cost-effective and environmentally friendly way to increase nutrient supply inside the soil ecosystem (Shridhar, 2012; Philippot, 2013). Other tropical diazotrophic bacteria, such as *Azospirillum*, have been investigated in Brazil for their practical application in corn, wheat, sugarcane, and rice, as well as used as co-adjuvants in soya beans, which have shown favorable positive outcomes (Hungria et al., 2010; Fernandes, 2014).

**TABLE 1:** Direct and indirect effects of microbiome on soil and its surrounding plants.

Plant growth-promoting microorganisms (PGPM)	Plant species	Direct and indirect effects on soil and its surrounding plants	References
*Pseudomonas* sp., *Enterobacter* sp.	*Oryza sativa*	Improved root architecture, lateral root outgrowth, seminal roots, root length, enzyme activities, nitrogen and phosphate uptake.	Chandarana et al., 2022
*Pseudomonas koreensis*, *Ralstonia pickettii, Bacillus cereus*	*Brassica oleracea*	Induced vegetative characteristics, antioxidant activities, phenolic compounds, nutrient acquisition, phytochemical composition and overall crop productivity.	Helaly et al., 2022
*Piriformospora indica*	*Trigonella foenum graecum*	Enhanced shoot and root length, shoot and root dry weight, leaf area, and number of leaves physiological responses, viz., photosynthetic rate, stomatal conductance, transpiration and internal CO_2_, and biochemical aspects like carotenoids, chlorophylls, nitrogen, and protein content.	Bisht et al., 2022
*Bacillus paranthracis* and *Bacillus megaterium*	*Solanum nigrum*	Stimulated Indole-3-Acetic Acid and salicylic acid levels.	Chi et al., 2022
*Bacillus cereus*, *Bacillus thuringiensis*, *Buttiauxella agrestis*	*Musa paradisiaca*	Increased levels of total height, pseudostem diameter, root length, number of leaves, fresh root mass, pseudostem, leaf area, stomatal conductance, internal carbon concentration, photosynthesis rate, transpiration rate, leaf temperature and chlorophyll.	Araújo et al., 2022
*Azospirillum brasilense, Gluconacetobacter diazotrophicus, Herbaspirillum seropedicae*, and *Burkholderia ambifaria*	*Allium cepa*	Increased plant height, total chlorophylls, crop yield, dry matter, phenolic contents, antioxidant activities, soil fertility, microbial community structure, total organic carbon, organic matter, and available phosphorus and nutrient levels.	Pellegrini et al., 2021
*Pseudomonas* sp., *Enterobacter* sp.	*Lycopersicon esculentum*	Enhanced metabolites such as flavonoids, carbohydrates, amino acids and carboxylic acids.	Zuluaga et al., 2021
*Bacillus*, *Brevibacillus*, *Agrobacterium*, and *Paenibacillus*	*Vicia faba*	Boosted growth-promoting activities such as phosphate solubilizing, ammonia production, extracellular enzymatic activities, indole-3-acetic acid, plant height, shoot dry weights, proline contents, enzymes activities and accumulation of mineral nutrients.	Mahgoub et al., 2021
*Bacillus subtilis*	*Zingiber officinale*	Stimulated plant height, leaf length, number of leaves per plant, leaf width and chlorophyll content.	Jabborova et al., 2021
*Bacillus pumilus*	*Oryza sativa*	Enhanced indole-3-acetic acid,1-aminocyclo propane-1-carboxylicacid (ACC) deaminase activity, P-solubilization, proline accumulation, exopolysaccharides production, chlorophyll, carotenoids, antioxidant soil enzyme activities, such as alkaline phosphatase, acid phosphatase, urease, and β-glucosidase.	Kumar et al., 2021
*Actinomycetes, Streptomyces*	*Triticum durum*	Higher shoot and root length, dry and ash-free dry weight, and the total chlorophyll content, as well as proline accumulation	Djebaili et al., 2021
*Pseudomonas reactans*, *Pantoea alli*, *Rhizoglomus irregulare*	*Zea mays*	Ion homeostasis, growth promotion, nutritional balance and crop productivity.	Moreira et al., 2020
*Bacillus cereus* TCR17, *Providencia rettgeri* TCR21 and *Myroides odoratimimus*	*Sorghum bicolor*	Induced synthesis of siderophores, metabolites, indole-3-acetic acid, phosphate solubilization, plant growth, antioxidant activities and decreased proline and malondialdehyde content oxidative damage.	Bruno et al., 2020
*Aneurinibacillus aneurinilyticus, Paenibacillus* sp.	*Phaseolus vulgaris*	Stimulated root length, fresh/dry weight, biomass and total chlorophyll.	Gupta and Pandey, 2019
*Curtobacterium albidum*	*Oryza sativa*	Higher nitrogen fixation, exopolysaccharide production, hydrogen cyanide, Indole-3-acetic acid, and 1-aminocyclopropane-1-carboxylate (ACC) deaminase activity along with plant growth parameters, photosynthetic pigment efficiency, membrane stabilization index and proline content and antioxidative enzymatic activities.	Vimal et al., 2019
*Bacillus subtilis*, *B. amyloliquefaciens*, *Pseudomonas fluorescens*, and *P. aeruginosa*	*Clavibacter michiganensis*	Siderophores, hydrogen cyanide and indole acetic acid production.	Abo-Elyousr et al., 2019
*Asprgillus fumigatus*, *Fusarium proliferatum*	*Oxalis corniculata.*	Growth promoting traits such as, siderophores production, phosphate solubilization, bioactive compounds, indole acetic acid and gibberellins synthesis.	Bilal et al., 2018
*Piriformospora indica*	Several plant species	Induced hormonal synthesis and signaling cascade for boosting growth, flowering, differentiation, immune responses	Xu et al., 2018
*Bacillus* sp.	*Capsicum annum*	Induced proline production and antioxidant enzyme activities, growth and development.	Wang et al., 2018

When all the soil components are balanced properly, such as C sources availability, aeration, oxygenation, acidity, alkalinity, and inorganic nutrients like N, P, and S, then, it causes the expansion of the phytomicrobiome variability and population and thus resulting in healthy and fertile soil (Johns, 2017; Wang et al., 2018). Therefore, the presence of phytomicrobiome in the form of archaebacteria, bacteria, and fungi inside the soil system causes the enhancement of soil fertility and soil health. Subsequently, it creates a healthy relationship between the soil-plants system, forming a suitable agricultural management strategy with more sustainability and eco-friendly nature (Pereira et al., 2021).

### Phytomicrobiome and abiotic stresses

Abiotic stresses play a major role in dwindling the agricultural productivity and yield. High light intensity, heavy metals, heat, chilling, drought, cold, salinity, nutrient, and ozone are the few abiotic stresses that affect the plants in different aspects (Suzuki et al., 2014). They are responsible for weakening the plant defense mechanism and make it more susceptible for other stresses (Mittler and Blumwald, 2010). Moreover, they impair growth, development, yields, and nutrient uptake in plants during stresses. And these characteristics, such as soil quality, nutritional status, and various physicochemical activities, play a major role for plant growth and development. The adverse effects due to stresses are faced by various agricultural crops globally that decline their overall productivity and yield output. The management strategies adopted against such constraints do not always follow the target-oriented criteria, yet the soil fertility gets affected due to excessive usage of fertilizers as well as pesticides that, no doubt, enhance the yield of the crop but also hinder environment and ecosystem, thereby worsening the living populations. The microbes, however, can be manipulated in agro-farming for best management of abiotic stresses along with stimulating crop yields.

In order to combat these stressful conditions, plant-microbe interactions serve to be one of the most effective mechanism to combat the adverse conditions. Rhizosphere is comprised of numerous microbes that mediate plant growth and are ecologically significant and exist either in free or symbiotic form. Many microbes, endophytic or free-living, stimulate plant growth in a direct or indirect manner (Shameer and Prasad, 2018). The microorganisms present in the rhizosphere help in increasing the plant yield by improving the soil health and root growth (Asati et al., 2016). Several microbial strains, such as *Frankia* sp., *Bacillus* sp., *Azotobacter* sp., *Pseudomonas* sp., *Burkholderia* sp., *Enterobacter* sp., *Bradyrhizobium* sp., *Kocuria* sp., *Flavobacterium* sp., *Alcaligenes* sp., *Serratia* sp., *Klebsiella* sp., *Microbacterium* sp., *Agrobacterium* sp., *Chromobacter* sp., and *Rhizobium* sp., are known for possessing growth-promoting traits (Sarkar et al., 2018; Shah et al., 2021). The plant-microbe associations are highly complex, and the fundamental step of this interaction is due to the secretions released by roots in the form of organic compounds that act as nutrients within the rhizosphere. Plants play a crucial role for assembling and colonizing rhizospheric entities for their own benefits (Zhalnina et al., 2018). Subsequently, the colonized micro-organisms enhance the overall plant metabolic activities during stresses through different mechanisms, namely, soil restoration and reclamation, synthesis of growth-promoting compounds, inhibiting pathogenic organisms, nutrient acquisition, and phosphate/nitrogen solubilization, respectively (Etesami and Adl, 2020; Kour et al., 2020; Liu et al., 2020). The basic mechanism adopted by microbes includes nitrogen fixation, phosphate solubilization, iron sequestration, and production of plant hormones (Chauhan et al., 2021). This is mainly accomplished by enhancing the metabolic pathways of plants during stresses, thereby reducing the adverse effect of stressors from the plants. Moreover, inducing nitrogen fixation, phosphate solubilization from soil is mediated by microbes at an effective rate in order to overcome any metabolic adversity. The nitrogen and phosphate requirement is fulfilled by the plant under stress conditions as mediated by microbes. Moreover, they also trigger iron sequestration so as to balance their levels in the plants. Alongside, the most important aspect is synthesis of phytohormones by microbes that play an important aspect to prevent from stresses. To illustrate, the salicylic acid and jasmonic acid pathway is induced as a defense strategy in plants to cope with the stresses. Also, indole-3-acetic acid, gibberellins, and abscisic acid are released by the microbes in plants to regulate the normal functioning and metabolic processes. Microbes in the rhizosphere of plants cause induction of 1-aminocyclopropane-1-carboxylic acid (ACC)-deaminase activity and systemic resistance against the pathogens, which help the plants to defend the stressful conditions (Benáková et al., 2017). Several reports depicting the role of microbes during abiotic stresses have been tabulated in Table 2.

**TABLE 2:** Mechanisms adopted by different microbes in plants for abiotic stress management.

	Microbes	Plant species	Mechanism of action	References
Heavy metal	*Azotobacter* sp., *Pitchia* sp.	*Lepidium sativum*	Lowered metal accumulation [Cr(VI) and Cd(II)]	Diaconu et al., 2020
	*Bacillus subtilis, Pseudomonas aeruginosa, Paenibacillus jamilae*	*Spinacea oleracea*	Improved plant growth by microbes under metal (Cd and Pb) stressed conditions	Desoky et al., 2020
	*Stenotrophoonas maltophilia, Agrobacterium* sp.	*Arundodonax*	Enhanced As detoxification	Guarino et al., 2020
	*Calendula officinalis*	*Claroideoglomus claroideum, Funneliformis mosseae*	Higher accumulation of secondary metabolites and improved antioxidant capacity of plants during metal (Pb and Cd) stress.	Hristozkova et al., 2016
	*Glomus fasciculatum and Pseudomonas putida*	*Helianthus annuus L.*	Improved plant growth, nutrient acquisition and decreased ROS production under Cd and Zn stresses.	Mani et al., 2016
	*Bradyrhizobium japonicum*	*Lettuce*	Enhanced root length, shoot length and biomass in plants due to increased production of IAA under Cd and Pb stress.	Seneviratne et al., 2016
	*Thiobacillus thiooxidans and Pseudomonas putida*	*Gladiolus grandiflorus L.*	Root length, shoot length, dry biomass of plant was increased along with the accumulation of Pb and Cd.	Mani et al., 2016
Drought	*Acinetobactercalcoacetiens, Penicillium* sp.	*Setaria italica*	Microbe-mediated stress alleviation and improved plant growth and development.	Kour et al., 2020
	*Trichoderma, Pseudomonas*	*Oryza sativa*	Enhanced antioxidative defense mechanism, growth and development.	Singh A. et al., 2020; Singh D. P. et al., 2020
	*Azospirilum brasilense, Bacillus* sp.	*Cercropiapa chystachya, Carinianaestrellensis*	Stimulated antioxidant defense with improved growth under stressed condition.	Tiepo et al., 2020
	*Sinorhizobium medicae*	*Medicago truncatula*	Boosted microbe-mediated root nodulation process with higher nutrient acquisition.	Staudinger et al., 2016
	*Pseudomonas libanensis TR1, Pseudomonas reactans Ph3R3*	*Brassica oxyrrhina*	Growth parameters were increased in terms of root growth, relative water content and pigments whereas proline and malondialdehyde content were decreased.	Ma et al., 2016
	*Pseudomonas putida MTCC5279 (RA)*	*Cicer arietinum L.*	ROS scavenging and induced expression of stress responsive genes.	Tiwari et al., 2016
	*Bacillus amyloliquefaciens 5113, Rhizobium leguminosarum (LR-30), Azospirillumbrasilense NO40, Mesorhizobiumciceri (CR-30 and CR-39), Azospirillum brasilenseNO40 and Rhizobium phaseoli MR-2*	*Triticum aestivum*	Growth, drought tolerance index and biomass of plant was improved due to exopolysaccharides, IAA and catalase activity.	Hussain et al., 2014
Salinity	*Pseudomonas reactans, Pantoea alli*	*Zea mays*	Improved plant growth after microbial inoculation.	Moreira et al., 2020
	*Bacillis cereus, Serratia marcescens, Pseudomonas aeruginosa*	*Triticum aestivum*	Enhanced overall growth and development of plants.	Desoky et al., 2020
	*Curtobacterium oceanosedimentum, Curtobacterium luteum, Enterobacter ludwigii, Bacillus cereus, Micrococcus yunninensis, Enterobacter tabaci*	*Oryza sativa*	Stimulated growth and development of plants and improvement of antioxidative defense system.	Khan et al., 2020
	*Piriformospora indica, Streptomyces* sp.	*Stevia rebaudiana* Bertoni	Growth and development of plant was improved.	Forouzi et al., 2020
	*Piriformospora indica*	*Trigonella foenum graecum*	Enhanced shoot and root length, shoot and root dry weight, leaf area, and number of leaves physiological responses, viz., photosynthetic rate, stomatal conductance, transpiration and internal CO_2_, and biochemical aspects like carotenoids, chlorophylls, nitrogen, and protein content.	Bisht et al., 2022
	*Bacillus tequilensis*	*Oryza sativa*	Increased indole-3-acetic acid, siderophore, exopolysaccharide, total protein, antioxidant enzyme activities.	Khan et al., 2021
	*Bacillus* sp.	*Oryza sativa*	Induced osmoprotectants, proline, sugars and antioxidant enzymes.	Shultana et al., 2021
	*Pseudomonas* sp.	*Cannabis sativa*	Improved morphological traits, and expression of stress-related genes.	Berni et al., 2022
	*Pseudomonas fluorescens*	*Daucus carota*	Enhanced synthesis of metabolites, morphological traits and nutrients.	Yadav et al., 2021
	*Piriformospora indica*	*Triticum aestivum*	High proline, tocopherol, carotenoids and antioxidant enzyme activities.	Singh and Tiwari, 2021

#### Drought stress

**FIGURE 3:**
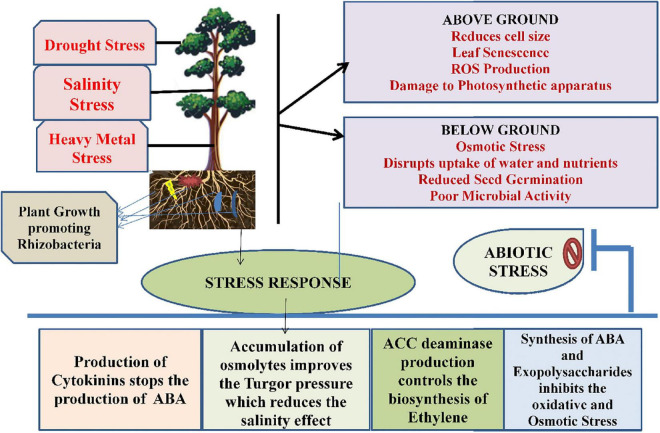
The role of plant growth-promoting rhizobacteria in association with plants under abiotic stress.

When plants are subjected to drought stress, it reduces size of the cell, damages membrane integrity, causes senescence of leaf, enhances the production of reactive oxygen species, thereby reduces the plant productivity (Tiwari and Rana, 2015). Studies conducted by Lata and Prasad (2011) found that drought stress impedes several physiological and molecular alterations within plants. It also alters pigment content and, therefore, damages the photosynthetic apparatus. Ethylene production is also increased in plants. Microbes are able to tolerate drought stress along with plant growth and development. During stress, these microbes form a thick wall, accumulate osmolytes synthesized exopolysaccharides or become dormant to combat the stressed conditions (Porcel et al., 2014). Several microbes playing a critical role under drought stress include *Azosprillum brasilense*, *Pseudomonas aeruginosa*, *Alcaligenes faecalis*, *Pseudomonas putida*, *Bacillus thuringeinsis*, etc. (Table 1; Naseem and Bano, 2014; Cohen et al., 2015; Ortiz et al., 2015). There are several direct and indirect mechanisms that are followed by microbes that include production of phytohormones as abscisic acid (ABA), Indole-3-acetoc acid (IAA), cytokinins, formation of bacterial exopolysaccharides, induced systemic tolerance, ACC deaminase production (Porcel et al., 2014), which has been shown in Figure 3. Studies conducted by Goswami et al. (2015) found that IAA under drought stress helps in the differentiation of lateral and adventitious roots, promotes root and shoot growth, and undergoes differentiation of vascular tissues. Similar to IAA, ABA also plays a very important role under drought stress through regulation of root hydraulic conductivity and, also, transcription of genes related to drought (Cohen et al., 2015). Bacteria also control the formation of ethylene by hydrolyzing ACC into ammonia and α-ketobutyrate with the help of bacterial ACC-deaminase (Bal et al., 2013). There are some microbes, such as *Pseudomonas aeruginosa*, *Proteus penneri*, and *Alcaligenes faecalis* that help to combat drought stress through the production of exopolysaccharides within maize plants. They help in increasing relative water content, sugars, and proteins through induction in proline content (Naseem and Bano, 2014). Similarly, combination of two microbes *Bacillus thuringiensis* and *Pseudomonas putida* assists in decreasing stomatal conductance and leakage of proline within the roots and shoots of plants (Ortiz et al., 2015).

#### Salinity stress

Salinity is also considered as an important abiotic stress for modern agriculture. Salinity is responsible for causing low water potential, eventually leading to osmotic stress, and plants are unable to take water and nutrients properly. These salts are present in soil as electrically charged ions (cations as Na^+++^, Ca^2+^, Cl^–^, NO_3_−, K^+^) and are generated due to weathering and inadequate rainfall (Shrivastava and Kumar, 2015). Apart from all these consequences, salinity stress also disturbs the ecological and physicochemical balance, affects seed germination, and reduces crop productivity and poor microbial activity as a result of negative effects of ions and osmotic stress (Shrivastava and Kumar, 2015). There are many microbes that are involved in salinity stress tolerance in plants and are tabulated in Table 2. Microbes use both direct and indirect mechanisms for the tolerance in plants. The direct mechanism involves production of phytohormones, nutrient mobilization, nitrogen fixation, siderophore production, and ACC deaminase by rhizobacteria. An enzyme named rhizobitoxine helps in inhibiting the production of ethylene (Hayat et al., 2010; Vardharajula et al., 2011). Reports from Vardharajula et al. (2011) showed that microbes help in salinity stress resistance by the accumulation of osmolytes in the cytoplasm that acts against the osmotic stress regulator, thereby maintaining the turgor pressure of cells and improving the plant growth. Production of exopolysaccharides by microbes also helps in binding onto cations and, therefore, makes them unavailable to plants under stress conditions and facilitates plant growth. The bacterial strains, *Bacillus pumilus* and *Bacillus subtilis*, also stimulate defense mechanisms under salinity stress through production of IAA, hydrogen cyanide, ammonia, and phosphate solubilization, respectively (Damodaran et al., 2013).

#### Heavy metal stress

Advancement in the agricultural practices and industrialization have resulted in heavy metal contamination in soil. Due to increasing heavy metals in soil, the productivity of crops is declining and affecting the growth and development of the plant (Ma et al., 2016). Among all the heavy metals, Pb is widespread and is known to cause root growth inhibition due to impairment of cells near root tips (Zia et al., 2020). Cr toxicity also causes injury to roots and leads to chlorosis of leaves. Similarly, Cd in rhizosphere is also harmful as it causes browning of roots, injuries to shoots and roots, and chlorosis of shoots (Boo et al., 2011; Li et al., 2011). Due to change in climatic conditions, temperature also acts as a stressor for plants in the form of heat and cold conditions. Change in water content, plasma membrane, enzyme functioning, growth of plants, photosynthetic activity, and cell division are some of the toxic effects that are produced by the temperature stress (Alam et al., 2017). Both high- and low- temperature conditions act as stress for plants. In high temperature, the interactions of the roots with microorganisms in the rhizosphere are affected adversely, whereas nitrogen fixation and the nodulation process are adversely affected during low-temperature conditions (Grover et al., 2011). There is a wide variety of microbes that are used for amelioration of heavy metals stresses in plants, such as rhizobacteria and mycorrhizae, and many reports are tabulated in Table 2. The mechanism used by these microbes includes impermeability to metals, the efflux mechanism, metal complexation, EPS sequestration, enzymatic detoxification, and volatilization. Other than these processes, these microbes promote plant growth by producing IAA, ACC deaminase, and reducing ethylene concentration (Glick, 2010). Microbes also cause the bioaccumulation of heavy metals, which also remove heavy metals from soil. Actinobacteria, Proteobacteria, and Firmicutes are reported to remove Mn, Pb, and As from soil (Zhang et al., 2015). The mechanistic approach of PGPRs under heavy metal stress has been shown in Figure 3.

### Phytomicrobiome and biotic stresses

Plant growth, development, and productivity are majorly affected by biotic stress factors like viruses, bacteria, fungi, arachnids, and nematodes. These pathogens disrupt the normal metabolism of plants and impede agricultural production. Globally, the crop yield is decreased by 21–30%, specifically the cereals crop is greatly affected by plant diseases (Savary et al., 2019). On account of climate change, variation in precipitation and increase in atmospheric temperature has led to the generation of new crop pests and diseases (Naamala and Smith, 2020). Pesticides and chemical fertilizers are not only of high cost, but they also degrade the soil quality and persistence in nature and pollute the environment. Also, due to long-term usage of these chemical fertilizers the pathogens-evolved resistance among them, so phytomicrobiome studies form a great sustainable background for the control of plant pests and diseases. Microbes serve as biocontrol agents for plant protection from biotic stress factors and also improve their nutrient uptake, growth, and health (Chouhan et al., 2021). Antagonistic activity of many rhizospheric microbes removes the infection of soil-borne plant pathogens. Plant growth-promoting rhizobacteria synthesize many organic compounds, phytohormones, siderophores, lytic and antioxidant enzymes, and also elevate the level of the stress-responsive genes in host plants for protection against pathogen attack and thus suppress the onset of the disease (Hashem et al., 2017). They produce antibiotics that inhibit the growth of phytopathogens, e.g., an antifungal activity of 2, 4-diacetyl phloroglucinol (2, 4-DAPG) produced by pigment-forming rhizobacteria that suppress the development of many fungal species (*Aspergillus niger*, *A. flavus*, and *Sclerotium rolfsii*) (Chauhan et al., 2021). Various antibiotics, such as pyrrolnitrin, zwittermicin-A phenazine-1-carboxylic acid, pyoluteorin, kanosamine, and pantocin, are produced by some PGPR strains (*Bacillus, Azospirillum, Rhizobium*, and *Pseudomonas* species) that enhance the immunity of plants against invaders and induce their mortality (Kenawy et al., 2019). Bacteriocin, a proteinacious toxin produced by many bacterial species, protects plants against phytopathogens. Pyocins produced by *Pseudomonas* species that are popular bacteriocins showed antagonistic effect against pathogenic strains of *Xanthomonas* and *Pseudomonas*, respectively (Fernandez et al., 2020). A gaseous compound HCN has been recognized as a biocontrol agent against plant diseases, which is produced by many free-living microorganisms (*Chromobacterium, Bacillus, Burkholderia, Actinomycetes, Pseudomonas*, etc.) (Banerjee et al., 2020). It is also reported that HCN enhanced the availability of phosphate indirectly and thus improved the nutrient uptake of plants (Rijavec and Lapanje, 2016). Different types of hydrolytic enzymes, such as proteases, amylases, lipases, cellulases, chitinases, and β-1, 3-glucanases, are produced by many rhizobacterial strains (*Streptomyces* sp., *Paenibacillus terrae, Bacillus, Paenibacillus*, and *Brevibacillus*), which degrade the cell wall and cause cell destruction of phytopathogens (Thampi and Bhai, 2017; Bhadrecha et al., 2020). Various Volatile Organic Compounds (VOCs) are generated by rhizospheric *Pseudomonas* sp. (L–Ala–L–Ala–L–Ala, 4-hydroxybenzoic acid, phosphonoacetic acid, and octamethylcyclotetrasiloxane), which inhibit the mycelial growth of harmful fungal strains (Kong et al., 2020). They also trigger plants to induce systemic responses against phytopathogens. It is reported that rhizobacterium *Bacillus cereus* triggers the expression of Induced Systemic Resistance (ISR), inhibiting the infection of Pseudomonas syringae in Arabidopsis (Niu et al., 2016). It is also evaluated that induced systemic response is enhanced by rhizobacteria Pseudomonas taiwanensis in anthurium infected with bacterium *Xanthomonas axonopodis* pv. *dieffenbachiae*, which induces polyphenol oxidase activity and synthesizes phenolic compounds at higher amounts (Dhanya et al., 2020). Several reports depicting the role of microbes during biotic stresses have been tabulated in Table 3.

**TABLE 3:** Mechanisms adopted by different microbes in plants for biotic stress management.

	Microbes	Biotic stress	Plant name	Mechanism of action	References
	*Bacillus cereus*, *Bacillus thuringiensis*, *Bacillus anthracis*	*Aspergillus niger*, *Aspergillus flavus*	*Berberis lycium*	Bioactive secondary metabolites for antimicrobial, antifungal, antioxidant, antitumor and anticancer activities.	Nisa et al., 2022
	*Trichoderma asperellum* and *Bacillus subtilis*	*Rhizoctonia* sp.	*Mentha spicata*	Higher dry biomass, polyphenols, rosmarinic acid and other special metabolites that improved growth and survival and reduced the incidence of infection.	Castro-Restrepo et al., 2022
	*Bradyrhizobium japonicum*	*Ralstonia solanacearum*	*Solanum lycopersicum*	Enhanced ethylene and abscisic acid production leading to induced systemic resistance in plants.	Chattopadhyay et al., 2022
	*Streptomyces albidoflavus*	*Athelia rolfsii, Fusarium oxysporum, Plectosphaerella ramiseptata, Sclerotinia sclerotiorum*, and *Verticillium dahliae*	*Foeniculum vulgare*	Inhibited fungal mycelium growth along with plant growth promotion.	Carlucci et al., 2022
	*Bacillus cereus*	*Fusarium oxysporum*	*Kalanchoe*	Antimicrobial compound synthesis and triggered expression of marker genes encoding jasmonic and salicylic acid and activated defense pathways with enhanced immunity.	Madriz-Ordeñana et al., 2022
1.	*Trichoderma* and *Piriformospora indica*	*Fusarium oxysporum*	*Cucumis sativus*	Reduced disease incidence and MDA content. Enhancement in antioxidant activity, chlorophyll a, b, carotenoids, and total soluble protein content.	Lava and Babaeizad, 2021
1.	*Acrophialophora jodhpurensis*	*Rhizoctonia solani*	*Solanum lycopersicum* L.	Suppression of root and crown rot disease by reducing disease index and induction of GPX, CAT, SOD and APX activities, phenolic, relative water contents, lignin accumulation and cell membrane stability.	Daroodi et al., 2021
1.	*Trichoderma harzianum* and *T. koningiopsis*	*Fusarium solani*	*Miahuateco chili*	High rate of development and growth was recorded and inhibition of radial growth of *F. solani* was also recorded.	Miguel-Ferrer et al., 2021
1.	*Trichoderma asperellum* TasT1	*Fusarium oxysporum*	*Arabidopsis thaliana*	Increase in root length, fresh weight and accumulation of defense-related enzymes to improve disease resistance.	An et al., 2021
1.	*Trichoderma harzianum* TRIC8	*Plasmopara halstedii*	*Helianthus annuus* L.	Metabolites produced by TRIC8 treated seedlings enhanced the hypocotyl length and reduced the sporulation density of pathogen	Özer et al., 2021
1.	*Acrophialophor ajodhpurensis*	*Alternaria alternata*	*Solanum lycopersicum* L.	Increased activity of antioxidant enzymes, phenolic contents and relative water contents, lignin accumulation, cell membrane stability, accumulation of superoxide (O2-) and hydrogen peroxide.	Daroodi et al., 2021
1.	Plant growth-promoting actinomycetes	*Fusarium oxysporum* f.sp. *radicis-lycopersici*, *Rhizoctonia solani*, *Pseudomonas syringae*, *P. corrugata*, *P. syringae* pv. *actinidiae*, and *Pectobacterium carotovorum* subsp. *carotovorum*	*Solanum lycopersicum* and *Daucus carota*	The diffusible and volatile compounds of actinomycete strains showed antifungal and antibacterial activity which suppress the infection of phyto-pathogens.	Djebaili et al., 2021
1.	*Pantoea*, *Pseudomonas*, and *Curtobacterium*	*Xanthomonas oryzae*	*Oryza sativa*	Suppression of bacterial blight disease through antagonistic effect of endophytic microbes producing phytohormones, lipolytic enzumes, ACC deaminase, nitrogen etc.	Yang et al., 2020
1.	*Cladosporium*, *Itersonillia*, and *Holtermanniella*	*Fusarium* sp.	*Triticum aestivum*	Endophytic microbes colonized in wheat spike increases species diversity and influence plant response by decreasing Fusarium head blight.	Rojas et al., 2020
1.	*Trichoderma brevicrassum* TC967	*Rhizoctonia solani*	*Cucumis sativus*	Root surface colonized hyphae of the strain TC967 induced the expression of pathogenesis-related genes (PR1, PR4, and PR5) and reducing the disease index with plant growth promotion.	Zhang and Zhuang, 2020
1.	*Trichoderma* spp.	*Rhizoctonia solani*	*Vigna unguiculata*	Reduction of the disease indices and induced plant growth *via* P solubilization, IAA and siderophore production.	Chao and Zhuang, 2019
1.	*Trichoderma isolates*, viz., *Trichoderma harzianum*, *T. asperellum*, *T. virens*, *T. virens*, and *T. virens*	*Pythium aphanidermatum*	*Solanum lycopersicum* L.	Suppression of damping off and root rot disease caused by pathogen *via* systemic defense response stimulated by conidia of *Trichoderma* which active peroxidase, polyphenoloxidase and chitinase activities and other defense enzymes.	Elshahawy and El-Mohamedy, 2019
1.	*Streptomyces thermocarboxydus*	*Fusarium*	*Solanum lycopersicum*	Increased photosynthetic efficiency resulting in enhanced chlorophyll and photosynthetic parameters.	Passari et al., 2019
1.	*Pseudomonas* sp. 23S	*Clavibacterm ichiganensis* subsp. *michiganensis*	*Solanum lycopersicum* L	Enhanced growth in root and shoot dry weight, higher content of mineral elements by inducing systemic response (ISR).	Takishita et al., 2018
1.	*P. fluorescens*, *Azospirillum brasilense*	*Alternaria* sp., *Curvularia* sp., and *Fusarium oxysporum*	*Oryza sativa*	Stimulated seedling and root hair growth.	Verma et al., 2018
1.	*Bacillus* sp.	*Pseudomonas* sp., *Xanthomonas* sp., and fungal pathogen (*Pythium* sp.)	*Solanum lycopersicum*, *Capsicum annum*, *Cucumis sativus*	Suppression of pathogens and improvement in growth	Liu et al., 2018
1.	*Pseudomonas putida* CRN-09 and *Bacillus subtilis* CRN-16	*Macrophomina phaseolina*	*Vigna radiata*	Enhanced levels of peroxidase, polyphenol oxidase, phenylalanine ammonia lyase, β-1,3 glucanase and chitinase activity resulting in increased plant immunity.	Sharma et al., 2022
1.	*Trichoderma atroviride* (TRS25)	*Rhizoctonia solani*	*Cucumis sativus*	Enhanced activity of guaiacol peroxidase, syringaldazine peroxidase, phenylalanine ammonia lyase, polyphenol oxidase and upregulation of ISR and SAR marker genes.	Nawrocka et al., 2018
1.	*Pseudomonas* sp. 23S	*Clavibacterm ichiganensis* subsp. *michiganensis*	*Solanum lycopersicum* L.	Reduced severity of bacterial canker by ISR using marker genes (PR1a salicylic acid, PI2 jasmonic acid and ACO ethylene).	Takishita et al., 2018
1.	*Bacillus subtilis* (BERA 71)	*Macrophomina phaseolina* (Tassi) Goid	*Vigna radiata*	Increased plant pigments, nutrients and antioxidant enzymes.	Hashem et al., 2017
0.	*Bacillus* sp. (CHEP5 specie) and *Bradyrhizobium* sp. (SEMIA6144)	*Sclerotium rolfsii*	*Arachis hypogea*	Reduced stem wilting incidence by increasing induced systemic resistance.	Figueredo et al., 2017

**FIGURE 4:**
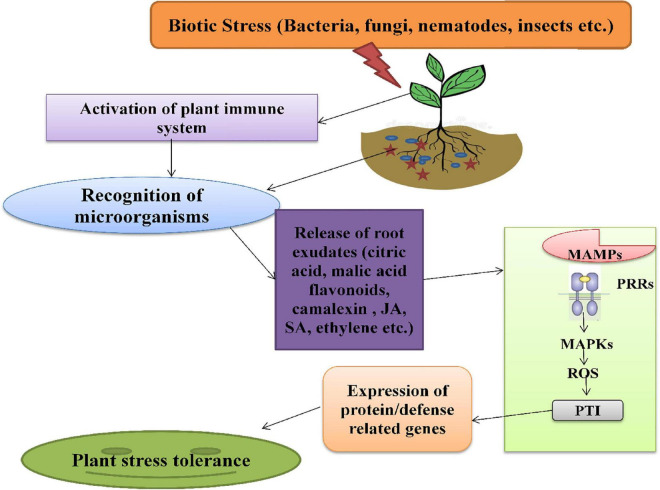
The mechanism of action of phytomicrobiome on plant immune system under biotic stress. MAMPs, microbe-associated molecular patterns; PRRs, pattern-recognition receptor; MAPKs, mitogen-activated protein kinases; ROS, reactive oxygen species; PTI, pattern-triggered immunity.

The plant immune system is activated during biotic stress, which triggers the roots to secrete several exudates, such as organic acids, phytohormones, alexins, etc. These exudates act as signaling components and are enhanced in response to various stresses. The complexes like polyphenols, plant hormones (JA, SA, and Ethylene), AHLs (N-acyl-homoserine lactones) are also involved in plants-microbes associations under stress severity (Bhatt et al., 2020). The AHLs molecules are produced by rhizospheric bacteria, which are then used for communication among themselves and, also, for regulation of genes in the signaling cascade. The root secretions attract various microorganisms that further activate the downstream signaling pathway of stress tolerance. Plants recognize rhizospheric microbes through the perception of MAMPs (microbe-associated molecular patterns) signals by PRRs (pattern-recognition receptors) located on plasma membrane. These high-affinity receptors activate MAPKs (mitogen-activated protein kinases) and generate ROS, followed by the activation of the pattern-triggered immunity of plants with enhanced expression of defense-related genes, respectively (PR genes) (Liu et al., 2020; Figure 4).

## Novel approaches for plant-microbiome communications: Metabolomics and metagenomics together with synthetic communities

**FIGURE 5:**
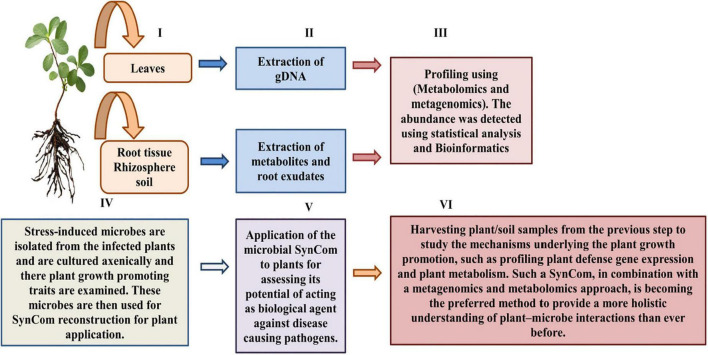
Integrated approaches for studying plant-microbiome communication research.

Until now, the majority of plant microbiome investigations have been found dependent on putative functions obtained from the genomic data, for instance, metagenomics (Liu et al., 2017). However, the recent application of metagenomics in combination with metabolomics and SynComs strategy has demonstrated its efficiency in overcoming the loopholes of plant-microbiome interaction studies (Castrillo et al., 2017; Berendsen et al., 2018; Durán et al., 2018; Kwak et al., 2018; Carrión et al., 2019). Basically, this integrated approach utilizes metagenomics to evaluate the structure and the probable role of plant-consociated microbiological metagenomes, proceeded by the isolation and culturing of fungus and bacteria for SynCom reconstruction (Durán et al., 2018). The SynCom constructed can thus be manipulated by its application to axenic plants (host plant deficient of its usual microbiota) for assessing various factors, for instance, to investigate microbial inter-kingdom interactions and, also, in determining their plant growth-enhancing potential as well as their effects on health and physiology of plants (Berendsen et al., 2018; Durán et al., 2018; Figure 5). Furthermore, by comparing stressed and non-stressed plant microbiomes, stress-induced microbiome alterations can also be observed. Thus, the stress-induced SynComs have been applied to plants in order to determine the biological significance of these modifications (Berendsen et al., 2018; Carrión et al., 2019). Moreover, the biochemical diversity of root exudates can be examined for changes in the presence or quantities of metabolites in order to discover how stress-induced microbiome construction is aided by root exudation. The major metabolic chemicals are isolated and evaluated *in vitro* for studying their interactions with plant microbial symbionts and pathogens, thus ultimately studying their impact on plant health. However, due to the chemical complexity of varied soil types, root exudation analysis remains an important hurdle in natural settings. A few plant compounds have been discovered in having a positive impact on the rhizosphere microbiota. These compounds, which include flavonoids, peroxidases, oxylipins, coumarins, benzoxazinoids, phenylpropanoids, aromatic chemicals, triterpenes, and mucilage, have all been shown to attract beneficial microorganisms that modulate plant defense system (Lombardi et al., 2018; Schulz-Bohm et al., 2018; Stringlis et al., 2018; Van Deynze et al., 2018; Huang et al., 2019).

Plant-associated microorganisms and metabolites as plant biocontrol agents could be investigated using the methods described above. Furthermore, in recent years, the ability of culturing bacteria for SynComs has substantially improved. As per the reports of Bai et al. (2015), more than 50% of *Arabidopsis thaliana*-associated bacteria may be isolated and conserved axenically. Isolation can be aided by isolation chips and selective media, such as imipenem-supplemented media, which select for a plant-growth-enhancer bacterium, *Stenotrophomonas maltophilia* (Bollet et al., 1995; Aleklett et al., 2018). SynComs built with culturable microorganisms provide defined, low-complexity consortia that may be used to investigate plant–microbe interactions in the lab. Due to the considerable intraspecies variability of bacterial genomes and the limited taxonomic precision of amplicon sequencing along with restricted databases, specific bacterial species/strains isolated from metagenomic investigations serve to be a significant problem. Complete-genome metagenomic sequencing with strain-level resolution may be able to reassemble whole bacterial genomes and overcome these limitations, but it comes at a higher cost and necessitates with advanced bioinformatic studies (Ranjan et al., 2016; Kwak et al., 2018; Carrión et al., 2019). Furthermore, when employing omics techniques and the SynComs approach, soil habitat-related characteristics (e.g., heterogeneity and edaphic features) that might significantly influence soil microbial function and composition should be taken into account (Young and Crawford, 2004).

## Phytomicrobiome-mediated mechanism in enhancing plant immune and defense system

Plant microbiome consists of a colossal range of microorganisms that are associated with various plant parts, be it leaf surfaces, pollens, nectar, and in the rhizosphere (Liu and Brettell, 2019). Plants recruit profitable microbes because of millions of year’s co-evolution. Hence, they offer enhanced approaches to fray both abiotic and biotic stressors and promote plant development by backing nutrient acquisition (Carrión et al., 2019). Root exudates largely influence the composition of microbes (Lombardi et al., 2018). It has been reported in maize that production of benzoxazinoids by roots attracts microbes that assist in strengthening plant defense (Kudjordjie et al., 2019). Likewise, under nitrogen (N)-deficit soils, leguminous plants discharge generous amounts of flavonoids to captivate N-fixing bacteria (Hassan and Mathesius, 2012).

**FIGURE 6:**
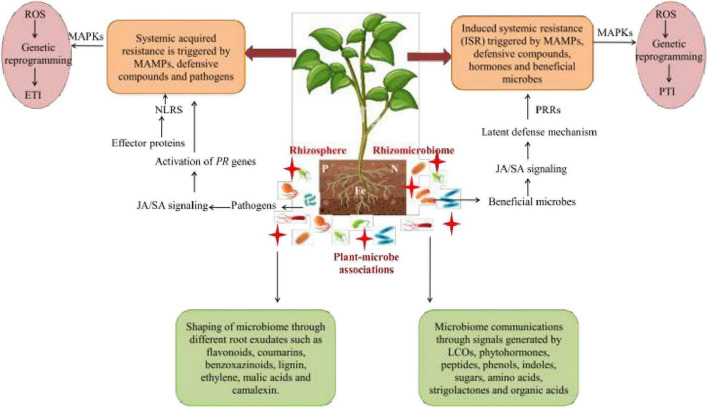
Microbe-mediated signaling mechanisms comprising of systemic acquired resistance (SAR) and induced systemic resistance (ISR) co-linked with plant defense system with the aid of signaling molecules. Beneficial microbes activate ISR that further activates ET, the SA/JA signaling pathway where pathogens stimulate SA levels and trigger SAR. The signaling cascade for immunity against stressors also activates the downstream signaling cascade by which effector proteins are detected by NLRs in order to activate MAPKs that generate ROS through genetic reprogramming for further activation of ETI. Moreover, plants also trigger defense processes to counteract stresses by MAMPs with the aid of PRR receptors in order to activate MAPKs for ROS generation by genetic reprogramming for further activation of PTI. SA, salicylic acid; JA, jasmonic acid; ET, ethylene; PR, pathogenesis; MAMPs, microbe-assisted molecular patterns; NLRs, nucleotide-binding leucine-enriched repeat receptors; MAPKs, mitogen-activated protein kinases; ROS, reactive oxygen species; PRR, pattern-recognition receptors; ETI, effector-triggered immunity; PTI, pattern-triggered immunity.

The interactions between plant immune system and microbiome are very complex (Teixeira et al., 2019; Figure 6). Pathogens compete with plant-associated microbes and then interact with plant innate immune system. It includes microbe-associated molecular patterns (MAMPs)-triggered immunity (MTI) and effector-triggered immunity (ETI) (Jones and Dangl, 2006). The nature of interactions between plant-associated commensals and plant immune systems is not definite, but it commands plant-stress interplay. Both pathogens and commensal microbes can trigger MTI and ETI. Their MAMPs are perceived by plant pattern-recognition receptors (PRRs) that reside in the plasma membrane (Teixeira et al., 2019). Nevertheless, commensal microorganisms benefit plants against stress by three means. (i) Lead to production of enzymes that assists in scavenging ROS (Yu et al., 2019) (ii) change MAMP structure that reduces plant immune responses (Teixeira et al., 2019) (iii) possess cell surface molecules that draw out plant defenses, i.e., T3SS and T4SS (Types III and IV secretion systems) (Levy et al., 2018).

The microbial communities play an important role to protect plants from insect herbivory and fungal pathogens (Elhady et al., 2018). Other than the normal soils, microbial composition plays an important role in disease suppression as it induces defensive gene expression in plants (Chialva et al., 2018). Many studies clearly validate that there is a connection between root microbiota and plant phenotypes (Zhang et al., 2019). Thus, rice variety having high nitrogen requirements has microbiome of greater nitrogen metabolic capacity than the variety having low nitrogen use (Zhang et al., 2019). Similarly, maize variety adapted to nitrogen-deficit soils has a plethora of diazotrophic bacteria to compensate for the nitrogen (Van Deynze et al., 2018). The microbiota also plays an important role in disease suppression as it is found that tomato variety resistant to *Ralstonia solanacearum* (causes tomato wilt) has rhizosphere rich in *Flavobacterium* spp. (Kwak et al., 2018). In *Arabidopsis*, a phosphate starvation response 1 (PHR1) was found to deal with phosphate stress responses and regulates a set of plant immune system genes, which ultimately lead to changing composition of root microbiome (Castrillo et al., 2017). Hence, ongoing studies are paving ways in understanding the role of microbiome in modulating plant defense, its survival, and growth in the future. Root microbiome has an impact on plant health by turning conductive soils into disease-suppressive soils and contributes to modulating plants’ immune system.

### Disease-suppressive soils

There is a tough competition among microflora populations in soils for available plant-derived nutrients (Raaijmakers et al., 2009). So, in order to infect the host, such soil-borne pathogens are required to maintain an intricately close association with host plants. To do so, they need to grow as saprophytes and in large numbers. Therefore, progress of the pathogen in infectious soils depends on the microbial community. The process of disease elimination in the company of microbial activity is termed as general disease suppression. The process of disease suppression turns highly productive due to organic amendments as it provokes activity of microbes. In order to understand biotic nature of specific disease suppression, the soils are refined (pasteurized) for removal of disease suppressiveness (Mendes et al., 2011).

### Development of disease suppressiveness

Soils can maintain their potential of disease suppressiveness for comprehensively long periods even if soils were left bare. Similarly, monoculture of a crop for several years improves soil suppressiveness (Bennett et al., 2012). The soil suppressiveness is developed against multiple crop diseases, such as in damping-off disease of sugar beet caused by *Rhizoctonia*, wheat diseases caused by *Gaeumannomyces graminis* var. *tritici*, *Fusarium* wilt disease of numerous plant species, and *Streptomyces* species causing potato scab disease, respectively (Mendes et al., 2011). To confer suppressiveness in conductive soils, microbes are isolated from suppressive soils followed by their inoculation into conductive soils. Raaijmakers et al. (2009) stated the role of microorganisms, such as *Proteobacteria* and *Firmicutes*, coupled with fungal members of Ascomycota to confer suppressiveness. The mechanism involved in order to bar soil-borne pathogen is through the production of lytic enzymes and antibiotic compounds (Doornbos et al., 2012).

### Beneficial rhizosphere microbes: Modulation of the host immune system

Many pieces of evidence were reported, stating the role of beneficial soil-borne microorganisms in boosting defensive capacity of aboveground parts of the plants (Zamioudis and Pieterse, 2012). Interestingly, the state of plants for triggered defense response is called induced systemic resistance (ISR) (De Vleesschauwer and Höfte, 2009). Furthermore, Millet et al. (2010) reported that root-associated *Rhizobacterium* WCS417 elicits production of low molecular weight molecules that subdue flagellin-triggered immune responses (Millet et al., 2010). Credible pieces of evidence state that systemic initiation of the immune signaling cascade aids in delivering resistance against a broad spectrum of pathogens and some insects (Pineda et al., 2010).

## Microbe-mediated molecular signaling cascade during stresses

Microbial signaling plays an essential role in regulating plant growth, as plants release organic compounds within the rhizosphere in the form of exudates that induce beneficial microbes. These microbes modulate plant behavior by generating inter-organism signaling molecules. The microbes are crucial for signaling, metabolism, and ion and hormonal homeostasis. They release antibiotic-like compounds to inhibit pathogens and induce nutrient acquisition during abiotic/biotic stresses in plants. Moreover, they initiate antibiotic production and elicit defense processes in plants. Signaling is governed by several molecules, such as N-acyl-homoserine lactones and volatile organic compounds, which play critical roles in downstream signaling and modulation of gene activities.

Nevertheless, cell-to-cell communication and signaling in plants take place through pathogen-associated molecular patterns (PAMPs) that enhance immunogenic responses in plants by identifying various chemical cues from microbes, and this immunity is called as PAMP-mediated immunity. In this process, the plants lead to callose deposits within the cell wall and ROS production with the activation of signaling and defense-related genes, such as *PR*-genes. For example, *PR-* genes, such as the ethylene-responsive gene (*ERF1*) and many other jasmonate-responsive genes (*LOX2, VSP, PDF*, etc.), behave as signaling molecules during stresses (Sharma et al., 2022). Furthermore, series of effector molecules are also released that generate another immune response in the form of effector-triggered immunity. This is an active defense process of plants, also called as gene-for-gene resistance and linked with resistance genes that are activated during stress alleviation. The resistance genes further regulate the expression of *PR* genes, NO accumulation, SA synthesis, oxidative burst, and programmed cell death, respectively (Wu et al., 2014). Along with this, many molecules are synthesized and transported *via* plant vascular system in order to behave as a mobile signal for activating a systemic acquired resistance (SAR) mechanism. SAR inhibits many pathogens and leaves prolonged effects through their mobile signals with the ability to activate the transcription of defense-related genes and plant immunity (Enebe and Babalola, 2019). SA has a predominant role in maintaining SAR (Figure 6). During incidence of pathogen infection, ISR is triggered by microbes along with the activation of genes encoding the SA-mediated defense signaling cascade for orchestration of plant defense responses (Ansari, 2018).

The interaction of plants with their proximity occurs through the virtue of some chemical signals that initiate from rhizodeposits, specifically from root exudations and mucilage secretions. And these chemical signals are in the form of primary or secondary metabolites (sugars, organic compounds, proteins, phenols, plant hormones, flavonoids, etc.). Under stresses, plants synthesize chemical cues in excessive amounts, like amino acids and sugars, when released in rhizodeposits and behave as chemo-attractants for beneficial microbes, causing enhancement in the microbial populations within rhizosphere. The mucilage secretions also consist of anti-microbial compounds that prevent the pathogen attack and establish mutual or symbiotic associations among plants and microbes (Basu et al., 2022). The leguminous plants also synthesize flavonoids and isoflavonoids, namely, naringenin and methoxychalcones that act as attractants for rhizospheric communities through regulating *nod* gene expression. Rhizobia also release lipochitooligosaccharides (LCO) that are mostly recognized by lysine-like receptors, thereby forming a symbiotic pathway to generate the nodulation (Basu and Kumar, 2020). The common symbiotic pathway signals are triggered by specific proteins called as common symbiotic proteins (CSP) that are widely present in nucleoplasm, plasma membrane, and nuclear membrane. Nucleoplasm contains three nucleoporins, cation channels, and core proteins. Others that are localized in cytoplasm are LysM-receptor kinases, leucine-receptor kinases, and NFR1/NFR5 (Basu and Kumar, 2020). The LCO signals generated by rhizobia form a special effect that act as signaling molecules (Clúa et al., 2018). Jasmonic acid also induces the expression levels of genes encoding LCO-biosynthesis, and LCO performs many functions within the plants, such as root nodulation, differentiation, etc. (Smith et al., 2015). In the similar fashion, the isoflavonoids also mediate regulation of nodulation genes (Liu and Murray, 2016). LCO also forms a communication mode during plant-mycorrhizal interactions, for example, strigolactone, a lactone homoserine is involved in plant-mycorrhizal signaling.

Apart from this, microbes also release a plethora of signaling compounds, such as antibiotics and plant hormones. To elucidate, thuricin 17 is a type of bacteriocin released by *Bacillus thuringenesis* that responds to plant signals and promotes plant growth along with inhibiting pathogenic organisms (Lyu et al., 2020). Moreover, various other compounds, such as lumichrome released by *Pseudomonas* sp. and *Sinorhizobium meliloti* and canavanine released by mucilage, are signaling compounds that favor in stress mitigation, root colonization, and symbiosis (Behm et al., 2014). The most probable mechanism by which the signaling molecules get activated is after reception of plant-microbe signaling during stresses, and this is called as positive regulation (Lyu et al., 2020). Contrastingly, the signaling molecules generated without any particular signal are known as negative regulation, and these signals are perceived through external/internal receptors present onto the cell wall. Here, the pattern recognition receptors (PRPs) are able to identify the microbial cell, followed by regulating the function of receptors, and, after activation, they initiate the signaling process to synthesize the crucial molecules (Bhatt et al., 2020).

## ‘Helping hand strategy’ for plant stress probiotics

Plants have developed a unique strategy to confront with stresses by attracting profitable microbes from environment to fight against that stress, which is referred to as Helping hand strategy (Liu and Brettell, 2019). This strategy can pave the way for the survival of future generations as it supports profitable plant-microbe interplay (Yuan et al., 2018). The helping hand strategy provides solution in different stress conditions. To combat pest/pathogen attack, various microbes, such as *Chryseobacterium* spp., *Chitinophaga* spp., *Flavobacterium* spp., and *Xanthomonas* spp., augment defense mechanisms in rhizosphere. They alleviate plant stress either by suppressing pathogen’s growth (Berendsen et al., 2018) or by activating the plant defense signaling pathway (Chen et al., 2018). There is an increase in plant and microbe symbiosis under phosphate/nitrogen starvation. They accumulate and provide the benefit under stress (Van Deynze et al., 2018). Hence, there exists a co-adaptive strategy between microbes and plants under peculiar stress (Terhorst et al., 2014). Different stressors in plants (metal toxicity, drought, poor nutrition) alter plant root metabolism and its microbial partners. Drought increases Actinobacteria in rhizosphere and/or in endosphere, which aids the plants to retaliate against drought conditions (Xu et al., 2018). Long distance signaling impelled by plant hormones plays a critical role in forming systemic-acquired resistance in plants (Pieterse et al., 2009). Application of hormones affects the microbial congregation in the rhizosphere, and bacterial forms recruited have potential to upgrade the plant defense responses (Lee et al., 2012).

**FIGURE 7:**
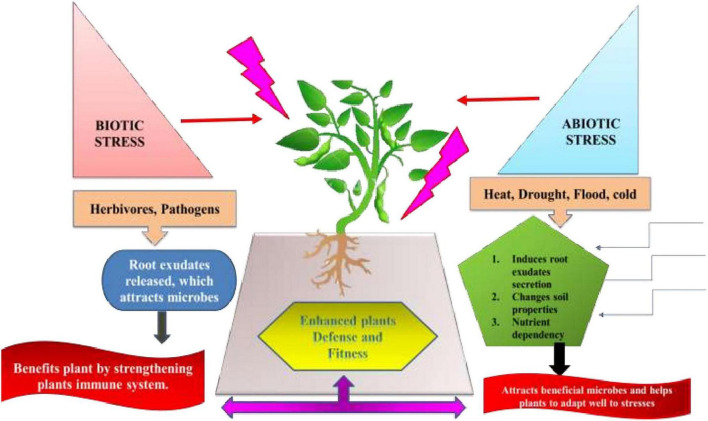
‘Defense Biome’ mechanisms favoring enrichment of particular microbes under diverse abiotic and biotic stressors by modulating root exudates composition, altering soil properties and nutrients availability.

Nevertheless, very less information is available for the impact of root and leaf stresses on phyllosphere microbiomes (Hammerbacher et al., 2019). There are pieces of evidence stating the role of plant volatile emissions in phyllosphere. Although data regarding influence of disease-induced changes on plant microbiome is inadequate but research is still going on for further revelations (Farré-Armengol et al., 2016). Many mechanisms need to be traced that are involved in the ‘Helping hand strategy,’ detecting the metabolites that play a critical role in interacting stressed plants and microbiome. There underlie many mechanisms that increase abundance of these profitable microbes under biotic and abiotic stresses. This phenomenon is termed as a “Defense Biome” mechanism (Figure 7). Under herbivore attack, the leaf and root exudates profile alters, resulting in attracting beneficial bacteria and/or fungi. Similarly, *Arabidopsis* roots secrete high amounts of citric acid in order to invite favorable bacterium *Bacillus subtilis* into the rhizosphere (Canarini et al., 2019). Furthermore, these microbes secrete quorum-sensing quenching molecules and antimicrobial compounds to obstruct pathogen growth and virulence (Raaijmakers and Mazzola, 2012). Both bacteria and fungi can have mutualism, where a bacterium supports fungal spore germination and, in turn, fungi provide nutrients and physical support (e.g., biofilms) for bacteria (Hoffman and Arnold, 2010). Likewise, abiotic stress (heat, drought, salinity, and mineral toxicity) led to abundance of plant-associated microbial commensals by (a) altering the root exudates profile and supporting growth of plant-associated microbes (b) changes soil properties, such as pH and nutrients levels (Schimel et al., 2007). Hence, the stress-induced microbial increase (*Stenotrophomonas* spp., *Pseudomonas putida*, and *Chryseobacterium* indologenes) is favorable to plants in enhancing host disease resistance (Neal et al., 2012; Santhanam et al., 2015).

## Artificial microbes against stresses

Artificial microbes are also called as synthetic microbes that form a novel concept for synthetic biology. It is highly useful for remodeling the plant microbiome by modulating its structure as well as function in order to maximize the plant benefits (Arif et al., 2020). There are various steps that are required for preparing efficacious artificial microbes. The initial step includes the determination of microbe and its origin followed by its procurement and cultivation according to its requirements. The next step includes the stimulation of microbial interactions according to their affinities and monitoring the efficiency of the artificially constructed consortium (Kong et al., 2018). With the ability of the plant microbiome to modulate the plant growth and metabolism by synthesizing phytohormones, Tsolakidou et al. (2019) designed two microbes with the ACC-deaminase activity. And inoculation of these microbial strains in *Lycopersicum esculentum* plants leads to lower the incidence of *Fusarium oxysporum* infection. Therefore, in conclusion, the microbial strains are best alternatives for stimulating plant growth during biotic and abiotic stresses. They are able to fill the lacunae for using conventional biofertilizers by resolving the issues related to environmental adjustment, host incongruity as well as futile competitive nature along with the native microbes (Hart et al., 2018). Certain microbes, namely, *Trichoderma* spp., are classified into Microbial Biological Control elements by Plant Protection Products agency (Woo et al., 2014). Furthermore, a plethora of these microbes are also categorized as biopesticides with the potential to enhance the plant growth and development (Lorito and Woo, 2015). Likewise, the arbuscular mycorrhizae, also known as biostimulants, prevent plants from pathogenic attack and other harmful organisms and diseases through activating an induced systemic resistance mechansim (Rouphael et al., 2015). Therefore, such examples elicit the need for registering the novel microbial consortium in order to counteract stresses and able to provide benefit toward plants. This would further boost the effective usage of microbes in the agriculture sector in the form of biopesticides, biofertilizers, biocontrol agents, and biostimulants (Woo and Pepe, 2018).

## Challenges and emerging solutions

Phytomicrobiome acts as a stable and effective alternative implication for replenishing the crop yield. It has been studied that microbial products have varied and unreliable effects during trials in different climatic conditions. However, the phytomicrobiome technology has potential, but there was a gap of understanding between the selection of microbes (native or inoculated strains) and its application. Due to the complexity in the mechanism of interactions among soil, plants, and environment, there was difficulty in persisting this phytomicrobiome study because only limited communications have been studied under naive conditions. Many industries showed interest in the use of microbial products that contain specific microbes and carrier-dependent inoculant strains. The implication of emerging technology “Microbiome on a Chip” was effective in studying the multitrophic interplay (microbiome plants) through the combinations of microbial colonies, the response of the host, and several environmental incentives (Stanley and van der Heijden, 2017). Advanced and facile technologies that have prolonged shelf lives would be a gainful strategy in the enhancement of the efficacy of microbial products. The evaluation and activities of the potential or native strains of microbes were helpful in monitoring the harmless microbes in field studies.

The enhancement in the production of goods was achieved by modulating the beneficial microflora of the soil at the blooming phase of seeds. This technology introduced harmless microbial strains and protected the plant biome from other competitive strains that further enhanced the survival and colonization of inoculated microbial strains. The chain of multiple harmless strains of microbes has more compatibility as compared to single-strain designed experiments because the networking and the grouping of beneficial strains with the existing microflora of soil favor the host. There were various systematic approaches to isolating microbial strains that are associated with phyllo- and rhizosphere (Bai et al., 2015; Mitter et al., 2017; Wallenstein, 2017).

Some reports depicted the emerging solutions to harness the ability of beneficial strains of microbes through *in situ* engineering of the microbiome for the profit in the agriculture and food sector. In this technology, the crops probiotics formulation was composed of the engineered microbiome that further manipulated the microflora (soil and plants) and its activity during its application (Mueller and Sachs, 2015; Sheth et al., 2016). Engineered strains affected the physiology of crops and microbiome by secreting the chemicals that effectively stimulated the activities of beneficial microbial strains, thereby providing resistance to crops against stresses. Based upon the phenotypic and genetic makeup of the microbiome, it was selected for the modulation in plants and soil native microbiome. Another proposed approach, domestication, was used for the elimination of traits facilitated by the microbiome through spraying of fertilizers, insecticides, and growth hormones for increasing crop productivity (Pérez-Jaramillo et al., 2016). The application of eco-friendly biofertilizers that contained beneficial microbial strains was an emerging solution for various crops and soil productivity. The genetic basis for interaction between plants and microbiome was understood by breeding microbe-enhanced designed plants during breeding programs, which, in turn, maintained the harmless strains of microbes.

Plants secreted specific chemicals (exudates) that engineered their own microbiome and enhanced the interactions with other beneficial microbiomes. It was reported that the miRNA was associated with maintaining the structural property of the microbiome of the rhizosphere. Therefore, these interactions are defined as a key to allocating the miRNA and engineering the beneficial microbiome from the target to beneficiary soil for desirable products. Different experiments were conducted with engineered soil or plant-enhanced microbes to manage the ecological engineering of the microbiome of plants and soil in an artificial selection system (Archana et al., 2020). This artificial ecosystem selection of microbial strains helped in improving the fitness of plants, resulting in evolving microbiome. *In situ* engineering of the genome was an effective and specific technology for the manipulation of the microbiome at a certain level. Another solution for enhancing disease and stress resistance in plants includes the combination of both the plant hormones and improved microbiomes, which activates the defense responses of the microbiome. Moreover, the interaction of microbiome allocation with diverse species of plants can be proved by discovering functional overlapped core microflora of the different plant species (Sheth et al., 2016).

## Conclusion and future perspectives

Food security and sustainable agri-products will be a considerable challenge in the coming years for the growing population. The stressed conditions reduce the productivity and availability of food for the growing population that creates rigidity in sustainable farming. Therefore, for enhancing the sustainability and productivity of soil, the phytomicrobiome is being used as an imperative biological toolkit to help and improve agricultural soil flora and fertility. It is a forgoing resource that counteracts various stresses by enhancing the synthesis of phytohormones, enzymes, nutrients, etc. This toolkit has a solution of boosting plant resistance against stresses by supplementing beneficial microbial stress-tolerant strains in soil or plants. The interface of the plant-soil microbe will promote productivity and growth by enhancing the production of hormones, siderophores, and the antioxidative defense system. Another technique, i.e., the application of plant growth-promoting rhizobacteria will also support the development of internal and external resistance or tolerance in plants under stress conditions. The plant growth-promoting bacteria may be used as priming agents, in the form of biopesticides and biofertilizers or as a direct supplement. It is revealed that the rhizo-microflora play an important role in plant growth and health, and the plant itself manages the composition of microbiomes. This mechanism of selection and regulation of microbial strains by plants is mostly favored for their maturation that appears as a natural-picking pressure whenever plants need microbial help.

The microbes act as ecological engineers to cope with environmental stresses. Still, stupendous research will be needed in the field of sustainable agriculture approaches by knowing the durability and survival, commercial design, inconsistency, and host relevance in extensive conditions to meet full utilization of these approaches and provide food security demands in the future. Also, there are other challenges in interpreting plant or soil physiological data and multi-omics with appropriate validation *via* lab and field experiments. This approach of understanding plant immunity, physiology, genetics, and biome under stress conditions needs to be explored more for improving productivity and ecological sustainability. Moreover, the biological explanation of the mechanism of microbiome shifts induced by stress would also allow the secretion of chemicals in biomes against stress is still unclear. The interplay between plants and microbes is a complex mechanism that needs a thorough investigation as it is still in its initial stage of experiments. Scientific policy organizers and associates need to make a toolkit to identify the plant growth-promoting microbes that are stress tolerant, and, also, the effective strains should be verified to overcome the harmful impact of the stressed environment. Proper research and study will achieve the process of selection and engineering of phytomicrobiome to make them stress resistant, resulting in enhanced production and yield of crops. Plant-microbe interactions and biotic stress-induced alterations have been mostly shown in plants, but the mechanism associated with metabolic pathways of microbes is still yet to be explored. Research should be focused on the characterization and effect of the microbiome on the gene expression in plants, resulting in altering the plant functioning both at proteomics and genomics levels. The CRISPR-Cas9-based forward genetic screen is a comprehensive approach to explain the interactions of plant and microbiome that will be beneficial in the future. To expand the knowledge of plant-soil-microbes interaction, information at the genomic level and engineering of beneficial microbial factories should be explored.

## Author contributions

KK, RB, SK, ASh, PAh, PAl, and TA designed the outline and revised and finalized the draft. All authors contributed to the article and approved the submitted version.
